# Low occupancy rate of the pedicle screw in the vertebral body leads to upper instrumented vertebral fracture

**DOI:** 10.1038/s41598-020-67337-3

**Published:** 2020-06-24

**Authors:** Shin Oe, Yu Yamato, Tomohiko Hasegawa, Go Yoshida, Sho Kobayashi, Tatsuya Yasuda, Tomohiro Banno, Hideyuki Arima, Yuki Mihara, Hiroki Ushirozako, Koichirou Ide, Tomohiro Yamada, Yuh Watanabe, Yukihiro Matsuyama

**Affiliations:** 1 Department of Orthopedic Surgery and Division of Geriatric Musculoskeletal Health, Hamamatsu University School of Medicine, 1-20-1 Handayama Higashi-ku, Hamamatsu, Shizuoka 431-3192 Japan; 2 Department of Orthopedic Surgery, Hamamatsu University School of Medicine, Hamamatsu, Japan; 3 Department of Orthopedic Surgery, Hamamatsu Medical Center, Hamamatsu, Japan

**Keywords:** Experimental models of disease, Outcomes research, Spinal cord diseases, Risk factors

## Abstract

Upper instrumented vertebra (UIV) fracture in adult spinal deformity surgery leads serious complications, such as spinal cord injury in 0.5–0.8%. Although tip-apex distance is important for preventing screw cut-out in proximal femoral fracture surgery, this suggest that the screw occupancy rate for bone fragments is also important. The purpose of this study was to investigate how the occupancy rate of pedicle screws (ORPS) affects UIV fracture. Patients with UIV fracture 1 year after surgery were defined as the fracture group (F); others were defined as the no fracture group (NF). ORPS, cut-out of pedicle screw (PS), medications, and bone mineral density were evaluated. Significant differences (P < 0.05) between group F (n = 58) and group NF (n = 260) were observed in age (71 years old in group F and 65 years old in group NF), diabetes medication use (19% in group F and 4% in group NF), steroid drug use (10% in group F and 2% in group NF), and ORPS (70% in group F and 76% in group NF). The cut-off value of ORPS using receiver operator characteristic analysis was 73%. Multiple logistic regression analysis identified diabetes medication use (P = 0.026, odds ratio [OR] 4.0) and ORPS < 73% (P = 0.001, OR 3.6) as significant risk factors for UIV fracture. The surgeon can’t control use of diabetes medication. However, they can replace with longer PS when ORPS < 73% is detected on radiographs taken during surgery. Further studies will be needed to better elucidate it’s use.

## Introduction

Proximal junctional kyphosis (PJK) is one of the common complications in adult spinal deformity (ASD) surgery. It has been reported that it occurs in 66% of patients with ASD within 3 months after surgery and in 80% of patients within 18 months^1,2^. PJK and proximal junctional failure (PJF) are complications that occur relatively early after surgery. Cho et al.^3^ reported in their meta-analysis that, although the incidence rate of PJK is 6.0–61.7%, many patients do not exhibit clinical symptoms. PJF, meanwhile, has an incidence rate of 4–15% and is frequently associated with clinical symptoms^4–7^. In addition, PJF cause sometimes severe neurological deficits in the cases with upper instrumented vertebra (UIV) fracture (0.5–0.8%)^8–10^.

**Figure 1:**
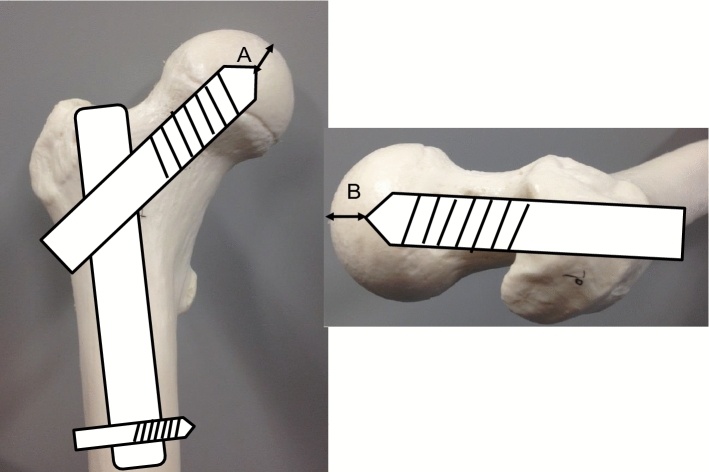
Tip-apex distance (TAD). TAD is the sum of the distance (**A** + **B**) from the tip of the lag screw to the apex of the femoral head on (**A**) an anteroposterior radiograph and (**B**) a lateral radiograph. It should be < 25 mm to prevent cut-out of the lag screw.

Baumgaertner et al.^11^ reported that failed fixation with a sliding hip-screw device in patients with proximal femoral fracture was related to the position of the lag screw in the femoral head. They concluded that the tip-apex distance (TAD) should be less than 25 mm to prevent cut-out of the lag screw. As shown in Fig. 1, TAD is the sum of the distance (A + B) from the tip of the lag screw to the apex of the femoral head on the (A) anteroposterior and (B) lateral radiographs. This result also suggests that the occupancy rate of the lag screw in the bone fragments is important for the prevention of cut-out. That is, in spinal fusion surgery, we made a hypothesis that the occupancy rate of the pedicle screw (ORPS) in the vertebral body is closely related to UIV fractures. Moreover, it was supposed that the way to set instrumentation such as the direction of pedicle screw (PS) or rod contour at the UIV is also related to UIV fracture. However, there are few reports about the relationship between how instruments are installed into the UIV and UIV fractures. In particular, there are no reports that investigate the relationship between the ORPS in vertebral bodies and UIV fractures. The purpose of the present study was to investigate the risk factors of UIV fracture (such as age, bone mineral density, and comorbidity) including the way in which instruments are installed in the UIV.

## Results

**Figure 2:**
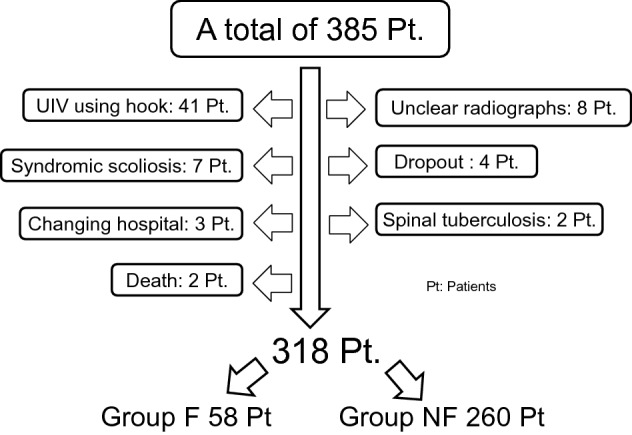
Recruitment flowchart.

A total of 67 patients were excluded from the study for the following reasons: instrumentation at the UIV was a transverse process hook (41 patients), unclear radiographs at 1 year after surgery (8 patients), syndromic scoliosis (7 patients), dropout from follow-up (4 patients), patient had follow-up at a different hospital (3 patients), spinal tuberculosis (2 patients), or death within 1 year after surgery (2 patients). Finally, 318 patients were evaluated in this study (Fig. 2).

Table 1 shows the characteristics of the patients in each group. Fracture group (F) and no fracture group (NF) contained 58 patients and 260 patients, respectively. The incidence of UIV fracture was 18.2%. Revision surgery (extension of fusion to cranial side of the vertebra) for UIV fracture was performed on 9 patients (15.8%) within 1 year after surgery. There were significant differences in age (70.8 years old in group F and 64.7 years old in group NF, P < 0.001), Lower instrumented vertebra [LIV] (18.6 in group F and 18.1 in group NF, P = 0.042), use of diabetes medication (19.0% in group F and 3.8% in group NF, P = 0.006), and use of steroid drugs (10.3% in group F and 1.9% in group NF, P = 0.046). T-scores for bone mineral density were determined for 37 of 58 patients in group F and 136 of 260 patients in group NF.

**Table 1:** Characteristics of patients.

	Fracture (F)	No fracture (NF)	P value
Number of patients	58 (18.2)	260 (81.8)	
Age (years)	70.8 ± 6.4	64.7 ± 14.0	0.000***
Female (%)	46 (79.3)	215 (82.7)	0.545
T-score (F:NF = 37:136)	− 1.7 ± 1.5	− 1.5 ± 1.1	0.501
UIV	9.1 ± 2.0	9.0 ± 2.4	0.654
LIV	18.6 ± 1.4	18.1 ± 1.6	0.042*
Osteoporosis drugs (%)	27 (46.6)	91 (35.0)	0.103
Mental disease drugs (%)	5 (8.6)	31 (11.9)	0.474
Diabetes drugs (%)	11 (19.0)	10 (3.8)	0.006**
Anti-rheumatic drugs (%)	2 (3.4)	8 (3.1)	0.884
Steroid drugs (%)	6 (10.3)	5 (1.9)	0.046*
Cancer drugs (%)	1 (1.7)	2 (0.8)	0.498
Anti-Parkinson drugs (%)	8 (13.8)	14 (5.4)	0.083

Data are presented as n (%) or mean ± standard deviation.

A numerical value has been attributed to each vertebral level as follows: 1 = T1, 2 = T2, 12 = T12, 13 = L1, 17 = L5, 18 = S1, 19 = ilium.

*UIV* upper instrumented vertebra, *LIV* lower instrumented vertebra.

*P < 0.05; **P < 0.01; ***P < 0.001.

The parameters related to instrument installations in the UIV are shown in Table 2. There were significant differences in the absence of TK of 30°–39° (94.8% in group F and 82.3% in group NF, P = 0.001), tip-apex distance of the PS [TAD, c] (10.7 mm in group F and 8.4 mm in group NF, P < 0.001), TAD[d] (10.5 mm in group F and 8.9 mm in group NF, P = 0.003), total TAD [a + b + c + d] (37.1 mm in group F and 32.9 mm in group NF, P = 0.015), anteroposterior diameter of the vertebral body at the UIV [APD] (34.9 mm in group F and 36.2 mm in group NF, P = 0.029), and ORPS (69.7% in group F and 76.2% in group NF, P < 0.001).

**Table 2:** CT scan and radiographic parameters.

	F (n = 58)	NF (n = 260)	P value
Cut-out of screw at UIV (%)	5 (8.6)	40 (15.4)	0.224
Not TK of 30°–39° (%)	55 (94.8)	214 (82.3)	0.001**
TAD; a (mm)	7.4 ± 3.9	7.1 ± 5.3	0.650
TAD; b (mm)	8.5 ± 4.3	8.6 ± 8.2	0.963
TAD; c (mm)	10.7 ± 3.6	8.4 ± 3.9	0.000***
TAD; d (mm)	10.5 ± 3.5	8.9 ± 3.9	0.003**
Total TAD; a + b + c + d (mm)	37.1 ± 9.0	32.9 ± 12.4	0.015*
APD (mm)	34.9 ± 3.8	36.2 ± 5.0	0.029*
ORPS (%)	69.7 ± 8.2	76.2 ± 9.5	0.000***
RVA (°)	− 3.4 ± 4.7	− 3.0 ± 5.6	0.595
SVA (°)	3.4 ± 4.0	2.2 ± 4.5	0.071

Data are presented as n (%) or mean ± standard deviation.

*UIV* upper instrumented vertebra, *TAD* tip-apex distance of pedicle screw, *APD*, anteroposterior diameter of vertebral body at UIV, *ORPS* occupancy rate of pedicle screw, *RVA* angle between rod contour and posterior wall of vertebral body, *SVA* angle between the direction of PS and upper endplate of UIV.

*P < 0.05; **P < 0.01; ***P < 0.001.

### The cut off value of ORPS

**Figure 3:**
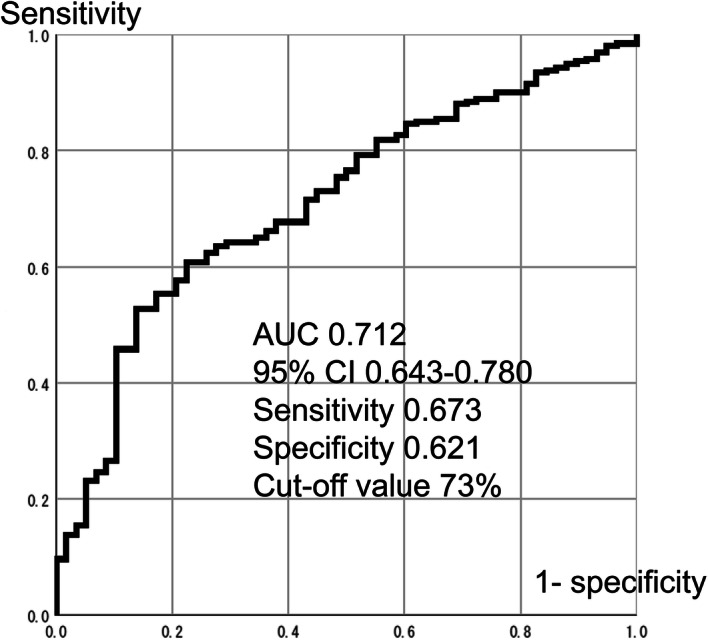
Cut-off value of ORPS using receiver operating characteristic curve. The cut-off value was 73% (area under the curve 0.712, 95% confidence interval 0.643–0.780, sensitivity 0.673, specificity 0.621). *ORPS* occupancy rate of pedicle screw.

The cut off value of ORPS was calculated using receiver operating characteristic (ROC) curves (Fig. 3). The cut off value was determined to be 73% (area under the curve 0.712; 95% confidence interval [CI] 0.643–0.780; sensitivity 0.673; specificity 0.621).

### The risk factors of UIV fracture

The significant risk factors of UIV fracture were assessed using multiple logistic regression analysis (Table 3). The dependent variable was set as the presence or absence of UIV fracture. The independent variables included age > 70 years, LIV level, use of diabetes medication, use of steroid drugs, ORPS < 73%, and absence of preoperative TK of 30°–39°. The significant risk factors of UIV fracture were the use of diabetes medication (P = 0.026, Odds ratio 4.012, 95% CI 1.182–13.619) and ORPS < 73% (P = 0.001, odds ratio 3.598, 95% CI 1.660–7.798).

**Table 3:** Multiple logistic regression analysis.

Risk factor	P value	Odds ratio	95% confidence interval
Dependent variable: presence or absence of UIV fracture Independent variable: age over 70, LIV level, diabetes remedy, steroid drug, ORPS below 73%, and not preoperative TK of 30°–39°			
Diabetes remedy	0.026*	4.012	1.182–13.619
ORPS below 73%	0.001**	3.598	1.660–7.798

*UIV* upper instrumented vertebra, *LIV* lower instrumented vertebra, *ORPS* occupancy rate of pedicle screw, *TK* thoracic kyphosis.

*P < 0.05.

**P < 0.01.

### The cases with paraplegia due to UIV fracture

**Figure 4:**
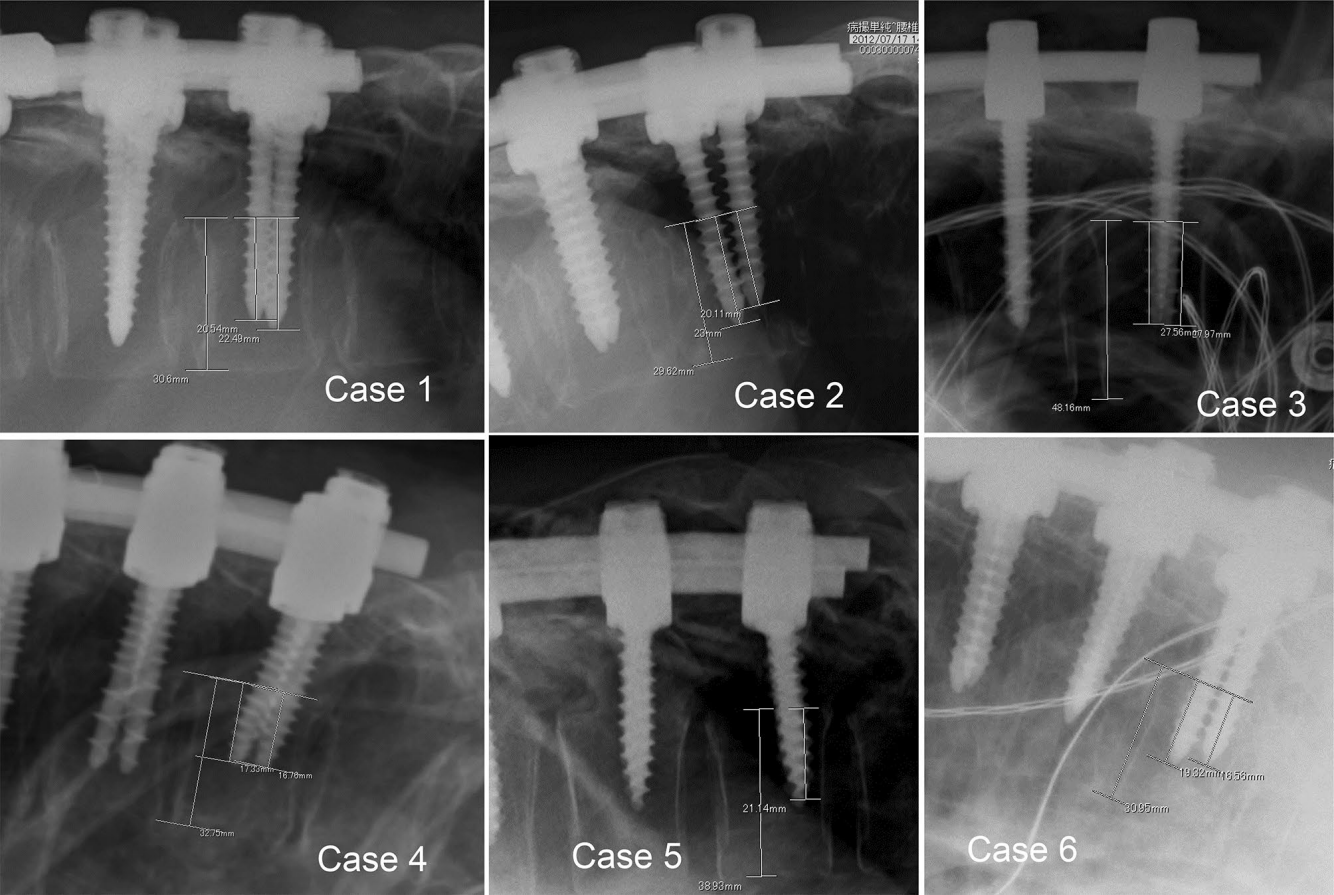
Radiographs in cases with paraplegia due to UIV fracture. All cases had ORPS < 73% (Case 1: 70.3%, Case 2: 72.8%, Case 3: 57.7%, Case 4: 52.0%, Case 5: 54.2%, Case 6: 63.9%). *ORPS* occupancy rate of pedicle screw, *UIV* upper instrumented vertebra.

Six of 58 patients with UIV fracture had severe paraplegia after ASD surgery. The details are shown in Table 4. Figure 4 shows intraoperative radiographs at UIV level in cases 1 to 6. Commonalities in these cases were (1) the LIV was the ilium and (2) ORPS < 73%. Moreover, many of them had complications that reduced bone strength (i.e. rheumatoid arthritis, diabetes, steroid drug, and hemodialysis). All of them were treated with extension surgery.

**Table 4:** The cases with paraplegia due to UIV fracture.

Case	1	2	3	4	5	6
Age	73	60	78	74	74	66
Sex	Female	Female	Male	Female	Male	Male
PMH	RA, DM steroid user	RA, DM, steroid user	Hemodialysis	Osteoporosis	Asthma	Parkinson
T-score	–	− 1.0	–	– 2.7	− 2.0	− 1.2
Trauma	–	+	+	−	−	−
UIV (initial surgery)	T5	T10	T9	T5	T10	T5
LIV	Ilium	Ilium	Ilium	Ilium	Ilium	Ilium
ASIA	D	B	C	B	C	D
ORPS (%)	70.3	72.8	57.7	52.0	54.2	63.9

*PMH* past medical history, *RA* rheumatoid arthritis, *DM* diabetes mellitus, *UIV* upper instrumented vertebra, *LIV* lower instrumented vertebra, *ASIA* American spinal injury association impairment scale, *ORPS* occupancy rate of pedicle screw.

## Discussion

Although many PJK cases do not exhibit clinical symptoms, PJF is often associated with clinical symptoms^3^. In particular, if fracture occurs at the UIV level, it may cause paraplegia. Therefore, it is extremely important to prevent UIV fractures. We determined that the risk factors of UIV fracture were LIV at the ilium level, use of diabetes medication, use of steroid drugs, absence of preoperative TK of 30°–39°, TAD, APD, and ORPS. Of these, age, use of diabetes medication or steroids, absence of preoperative TK of 30°–39°, and APD are dependent on the patient and therefore cannot be controlled by the surgeon. Conversely, selection of LIV and decisions regarding the length of the PS (TAD and ORPS) are factors that can be controlled by the surgeon. Nonetheless, LIV at the ilium level cannot be avoided in older patients with severe lumbopelvic deformity. The length of the PS in the UIV can be adjusted depending on radiographs taken during surgery. However, unlike the TAD in proximal femoral fracture surgery, the TAD in this study was greatly affected by the APD of the vertebral body. Thus, evaluation by ORPS is considered to be more appropriate because the vertebral body has a different APD depending on the spinal level. The incidence of UIV fracture was 11.1% (22 of 198) when the ORPS was 73% or more. On the other hand, when the ORPS was less than 73%, the incidence of UIV fracture was 30% (36 of 120). Lowe et al.^12^ reported in their biomechanical study that the mechanical strength at each region of the vertebral endplate was weakest at the center of the vertebral body. That is, when the ORPS is 73% or more, the tip of the screw is beyond the center of the vertebral body, which is considered to be effective for preventing UIV fracture. Additionally, multiple logistic regression analysis showed that significant risk factors for UIV fracture were the use of diabetes medication and ORPS < 73%. Janghorbani et al.^13^ reported that the risk of proximal femoral fractures increased 1.7-fold in patients with type 2 diabetes. Moreover, Yamamoto et al.^14^ reported that the risk of vertebral fracture in patients with type 2 diabetes increased 4.7-fold in males and 1.9-fold in females compared to non-diabetic participants. Diabetes is thus considered a strong risk factor for UIV fracture.

This study had several limitations. Firstly, it has been previously reported that the predominant risk factor of PJF was low bone mineral density^2^. However, we found no significant difference in the T-scores for bone mineral density between group F and group NF. These results might have been affected by the fact that not all patients had their bone mineral density examined (only 37 of 58 patients (63.8%) in group F and 136 of 260 (52.3%) in group NF). Secondly, we found that there was no significant difference in cut-out of the PS at the UIV. However, the number of PS cut-outs (on one side or both sides) was not evaluated because PS cut-out in both sides occurred in only one patient (1.7%) in group F and four patients (1.5%) in group NF. It will be necessary to evaluate this factor in a large scale study. Thirdly, even high ORPS cannot prevent vertebral fracture on the cranial side of the UIV. However, if there is no UIV fracture, the possibility of developing neurological symptoms is significantly decreased.

In conclusion, the significant risk factors of UIV fracture were the use of diabetes medication and ORPS < 73%. If radiographs indicate ORPS < 73% intraoperatively, it is important to replace the PS with a longer one to avoid UIV fracture.

## Materials and methods

### Ethical considerations

The study protocol was approved by the institutional review board of our university (Approval Number 14-306) and performed in accordance with Declaration of Helsinki. Informed consent was obtained from all participants.

### Patients

Four hundred and seventeen consecutive patients who underwent ASD surgery between April 2010 and January 2018 in our hospital were recruited retrospectively. ASD was defined as the presence of at least one of the following: degenerative or idiopathic scoliosis with cobb angle ≥ 20° in the coronal plane, Sagittal vertical axis ≥ 50 mm, pelvic tilt ≥ 25°, and/or thoracic kyphosis (TK) ≥ 60° in a standing radiograph of the whole spine^15^. The inclusion criteria were: (1) age ≥ 18 years, (2) follow-up > 1 year, and (3) four or more fused vertebra segments. The exclusion criteria were: (1) instrumentation other than PS (e.g., transverse process hook, sub-lamina taping) on one side or both sides of the UIV; (2) the presence of syndromic scoliosis; (3) infection; and (4) unclear radiographs. The patients were divided into fracture group (F) or no fracture group (NF). Group F was defined as patients with UIV fracture within 1 year after surgery, while Group NF was defined as patients without UIV fracture 1 year after surgery.

### Measured data

The following patient characteristics were evaluated: age, sex, bone mineral density, level of UIV and LIV, Cut-out of PS at the UIV, steroid use, and the use of any medications for osteoporosis, mental disease, diabetes, rheumatoid arthritis, cancer, or anti-Parkinson. The method of assessing was as follows:

- *Bone mineral density* measured using dual-energy X-ray absorptiometry for the total proximal femur and was expressed as a T-score.
- *The level of UIV and LIV* a numerical value was attributed to each vertebral level: 1 = T1, 2 = T2, 3 = T3, … 12 = T12, 13 = L1, 14 = L2, … 17 = L5, 18 = S1, 19 = ilium.
- *Cut-out of PS at the UIV* evaluated using postoperative computed tomography scans and was defined as the protrusion of the screw tip out of the vertebral body.

Preoperative standing X-ray of the whole spine was used to determine the presence or absence of TK of 30°–39°. We previously reported that the significant risk factor of PJK was TK over 40° or less than 30°^16^.

**Figure 5:**
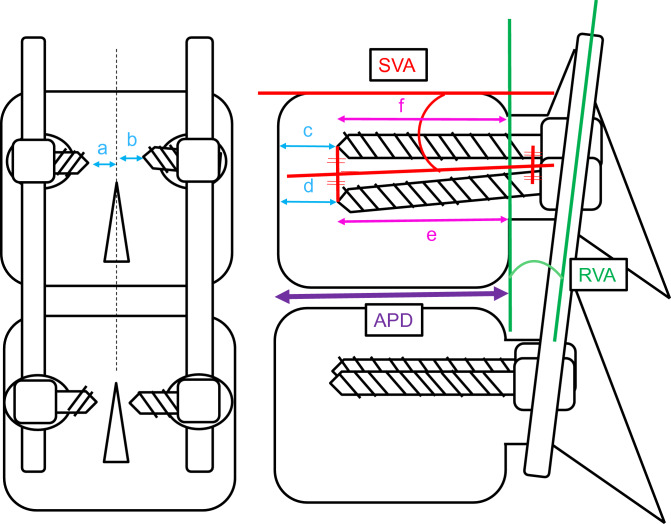
Parameters related to instrument installation at upper instrumented vertebra (UIV), TAD (Tip-apex and distance of the Pedicle screw [PS] [a + b + c + d]), APD (Anteroposterior diameter of vertebral body at UIV), ORPS (Occupancy rate of PS in upper instrumented vertebral body ([f + e) × 100/2 × APD). RVA (the angle between rod contour and posterior wall of vertebral body), SVA (the angle between the direction of PS.

As shown in Fig. 5, the parameters related to instrument installation at UIV were evaluated using radiographs taken during surgery. These parameters were as follows: TAD = [a + b + c + d], where a and b represent the distance from the tip of the PS to the center line of the UIV in the anteroposterior view, and c and d represent the distance from the tip of the PS to the anterior wall of the vertebral body in the lateral view, anteroposterior diameter of the vertebral body at the UIV (APD), ORPS in the UIV ([e + f] × 100/[2 × APD], where e and f represent the distance from the posterior wall of the vertebral body to the tip of PS); RVA (the angle between the rod contour and the posterior wall of the vertebral body, defined as positive when the tip of the rod leaves the vertebral body posterior wall), and SVA (the angle between the direction of the PS [the line from midpoint of tip to midpoint of base on the right and left PS] and the upper endplate of the UIV, defined as positive when the midpoint of the tip of the PS leaves the upper endplate of the UIV). UIV fracture was defined as a change of shape above grade 2 in the Genant classification^17^.

### Statistical analysis

All statistical analyses were performed using the SPSS version 23 statistical software package (IBM-SPSS, Inc., Chicago, IL, USA). Unpaired t-test was calculated to compare age, T-score, UIV, LIV, TAD, APD, ORPS, RVA and SVA between group F and NF. Chi-squared test were used to compare sex, use of medications, cut-out of PS at the UIV, and number of patients with TK of 30°–39°. The significant risk factors of UIV fracture were assessed using multiple logistic regression analysis. The cut off value of ORPS was calculated using receiver operating characteristic (ROC) curves. Statistical significance was set at *P* < 0.05.

### Ethical approval

The study protocol was approved by the institutional review board of Hamamatsu University School of Medicine, Shizuoka, Japan.
